# Interruption of Infection Transmission in the Onchocerciasis Focus of Ecuador Leading to the Cessation of Ivermectin Distribution

**DOI:** 10.1371/journal.pntd.0002821

**Published:** 2014-05-22

**Authors:** Raquel Lovato, Angel Guevara, Ronald Guderian, Roberto Proaño, Thomas Unnasch, Hipatia Criollo, Hassan K. Hassan, Charles D. Mackenzie

**Affiliations:** 1 Ecuadorian Onchocerciasis Program, Ministerio de Salud Pública, Quito, Ecuador; 2 Centro de Biomedicina, Laboratorio de Parasitología Molecular, Escuela de Medicina, Universidad Central del Ecuador, Quito, Ecuador; 3 Hospital Vozandes, Quito, Ecuador; 4 GHIDR Program, Department of Global Health, College of Public Health, University of South Florida, Tampa, Florida, United States of America; 5 Department of Pathobiology and Diagnostic Investigation, Michigan State University, East Lansing, Michigan, United States of America; Centers for Disease Control and Prevention, United States of America

## Abstract

**Introduction:** A clinically significant endemic focus of onchocerciasis existing in Esmeraldas Province, coastal Ecuador has been under an ivermectin mass drug administration program since 1991. The main transmitting vector in this area is the voracious blackfly, *Simulium exiguum*. This paper describes the assessments made that support the decision to cease mass treatment.

**Methodology and Principle Findings:** Thirty-five rounds of ivermectin treatment occurred between 1991–2009 with 29 of these carrying >85% coverage. Following the guidelines set by WHO for ceasing ivermectin distribution the impact on parasite transmission was measured in the two vector species by an O-150 PCR technique standard for assessing for the presence of *Onchocerca volvulus*. Up to seven collection sites in three major river systems were tested on four occasions between 1995 and 2008. The infectivity rates of 65.0 (CI 39–101) and 72.7 (CI 42–116) in 1995 dropped to zero at all seven collection sites by 2008. Assessment for the presence of antibodies against *O. volvulus* was made in 2001, 2006, 2007 and 2008 using standard ELISA assays for detecting anti-Ov16 antibodies. None of total of 1810 children aged 1–15 years (between 82 and 98% of children present in the surveyed villages) tested in the above years were found to be carrying antibodies to this antigen. These findings were the basis for the cessation of mass drug treatment with ivermectin in 2009.

**Significance:** This fulfillment of the criteria for cessation of mass distribution of ivermectin in the only known endemic zone of onchocerciasis in Ecuador moves the country into the surveillance phase of official verification for national elimination of transmission of infection. These findings indicate that ivermectin given twice a year with greater than 85% of the community can move a program to the final stages of verification of transmission interruption.

## Author Summary

Onchocerciasis has been known to be endemic in the northwestern coastal riverine jungle areas of the country since the early 1980's. A mass drug administration program with ivermectin was implemented in 1991, and in recent years has included consistent twice a year treatment. The impact of this program, and progress towards eliminating the transmission of *Onchocerca volvulus* from the endemic zone, was assessed by studying entomological parameters at sentinel sites in 1995, 2000, 2004 and 2008 using PCR detection of infective larvae in the vectors (*Simulium exiguum* and *Simulium quadrivittatum*); the survey of 2008 showed that all of the collection sites had reached a level consistent with the interruption of transmission. Serological assessment of children and adolescents in 2001–2008 also showed that transmission had been interrupted. These findings support the contention that the Ecuadorian Program has reached the post-treatment surveillance phase of the elimination program; mass drug administration _of ivermectin was consequently stopped._ The factors contributing to this successful achievement include ivermectin coverage of consistently around 85% or greater since 1998, careful consideration and control of possible expansions of the endemic area through migration, and the maintenance of a strong community health programme.

## Introduction

Onchocerciasis was recognized in Ecuador some 30 years ago and extensive work during the 1980s identified the limits of the endemic focus in Esmeraldas Province in north west of coastal jungle area of the country [1], [2]. This was area of increasing population where residents were spreading along the various river systems, with the population in the endemic area by 2010 reaching around 26,000 people. The clinical disease resulting from infection with *O. volvulus* was described in the 1980's to be amongst the severest of all the American onchocerciasis foci with blinding disease and extensive onchodermatitis [3]–[5]. The vectors in the focus include *S. exiguum and S. quadrivittatum* [6]–[8], with *S. exiguum* being the most important, as it is a highly efficient vector for *O. volvulus*; *S.exiguum* has a vectorial competency comparable to forest cytotypes of *S. damnosum* sensu lato in terms of the percentage of flies developing infective stage larvae (L_3_s) and the numbers of L_3_ per fly [9]. The second vector species, *S. quadrivitattum*, is much less efficient due to the presence of a cibarial armature which damages microfilariae ingested during a blood meal.

In the 1980s the only approach to treatment and control of this infection was the removal of palpable nodules containing the adult worms; however this nodulectomy campaign did little to reduce the increasing prevalence of infection and did not reduce the increasing prevalence of clinical disease [10]–[12]. In 1991, as part of the global approach to control of onchocerciasis using the microfilaricidal drug ivermectin twice a year treatment was implemented in the endemic zone of Ecuador. This mass drug administration approach was aimed at eliminating the disease and its transmission, and the program and the monitoring activities have followed the international guidelines established by WHO [13].

The Ecuadorian program is based on a close relationship with the community and is supported by a strong health services approach, with constant communication with the field locations and with strong local support. Although the environment is a particularly difficult one in terms of access the national program has been able to maintain a constant link with the endemic population. This has been essential as concerns regarding migration and expansion of the focus have always been important questions. A number of the foci in Latin American onchocerciasis are found in isolated locations, and the Ecuadorian focus is also a relatively isolated jungle location, thus often making such drug interventions difficult to implement.

The assessment of success and progress towards control and elimination of transmission described here has been monitored through entomological and serological parameters [13]. Although clinical parameters are believed to be important indicators and provide useful programmatic information as to the progress towards control and elimination, entomology and serology have usually been used to define the actual transmission status of a program, and importantly to assist in any decision to cease mass drug administration. This present communication describes the entomological assessment of the Ecuadorian foci and the findings that led to the decision to cease drug treatment in this important Latin American focus of endemic onchocerciasis.

## Materials and Methods

### Ethical statement

The Ecuadorian onchocerciasis control programme is carried out under the auspices and full approval of the National Ministry of Health and is thus fully supported by the Government of Ecuador. The distribution of drugs, the collection of skin and blood samples, as well as the collection of vectors, has been approved and monitored by the National Ministry of Health, and considered as ethical, safe, and to be of benefit to the local people. The study complied with current national and international regulations and standards for biomedical research in human subjects; informed consent was requested and obtained from the parents of the children for whom serological sampling was done.

### Study area

**Figure 1:**
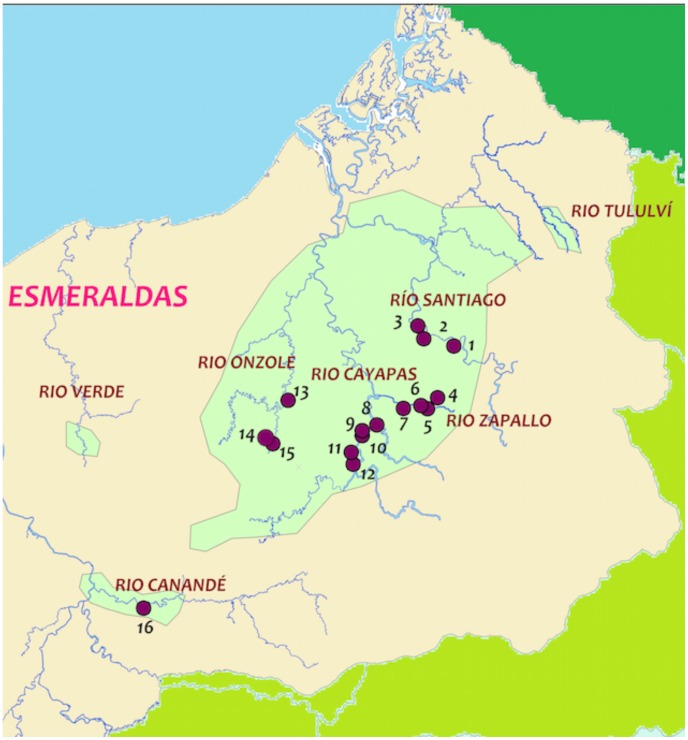
Map of the endemic area and locations of the entomological and serological surveys carried out between 1995 and 2008. 1- Playa de Oro, 2 - Angostura, 3 -Guayabal, 4 -Agua Salada, 5 -Pichiyacu, 6 - Playa Grande, 7- Herradura, 8 -San Miguel, 9- El Tigre, 10-Agua Blanca, 11- Callemansa, 12 - Corriente Grande, 13 – Colón de Onzole, 14 - Calpulí, 15 - Las Pavas, 16- Naranjal.

Ecuador is one of the six countries endemic for onchocerciasis in Latin America. The infection is found in a single focus in the Esmeraldas Province of Northwestern Ecuador, where it is associated with several river basins (Figure 1). The locations where the disease and infection is most prevalent being the three major river systems, Rio Cayapas, Rio Santiago and the Rio Onzole. A total of approximately 25,900 people were at risk in the focus in 2008. Coverage figures were maintained by a rigorous accounting system that is based on an active regularly updated census of the villages. Assessments for clinical presentation, ocular and skin parasite loads were carried out on a number of occasions pre-ivermectin treatment and during the treatement period and will be, or have been, reported elsewhere [11], [12]. In the hyperendemic area the main monitoring site on the Rio Cayapa had community microfilarial levels of 81% in 1982 which had increased to over 90% by 1987. When assessments were carried out in 2004 the dermal load had dropped to 4% and by 2008 all dermal biopsies were free of microfilariae. The last time microfilariae were identified in the eyes of any of the residents was in 2000.

### Drug distribution program

In 1987 Merck & Co. donated ivermectin for use in mass drug administration (MDA) programs for onchocerciasis [14]; Ecuador adopted this drug and began an MDA program in 1991. The Ecuadorian program has distributed drug once, or more usually twice, a year at 150 µg/kg to all eligible people (defined as those over 5 years of age, who are not pregnant or infirm); this is generally regarded to be approximately 80% of the total resident population. Drugs were distributed by local village health workers under the constant supervision of the Onchoerciasis National Program members from the central offices based in Quito and Borbon. The ivermectin distribution began in the main hyperendemic areas in 1991 with other communities added where necessary over the next two years.

### Drug coverage

**Figure 2:**
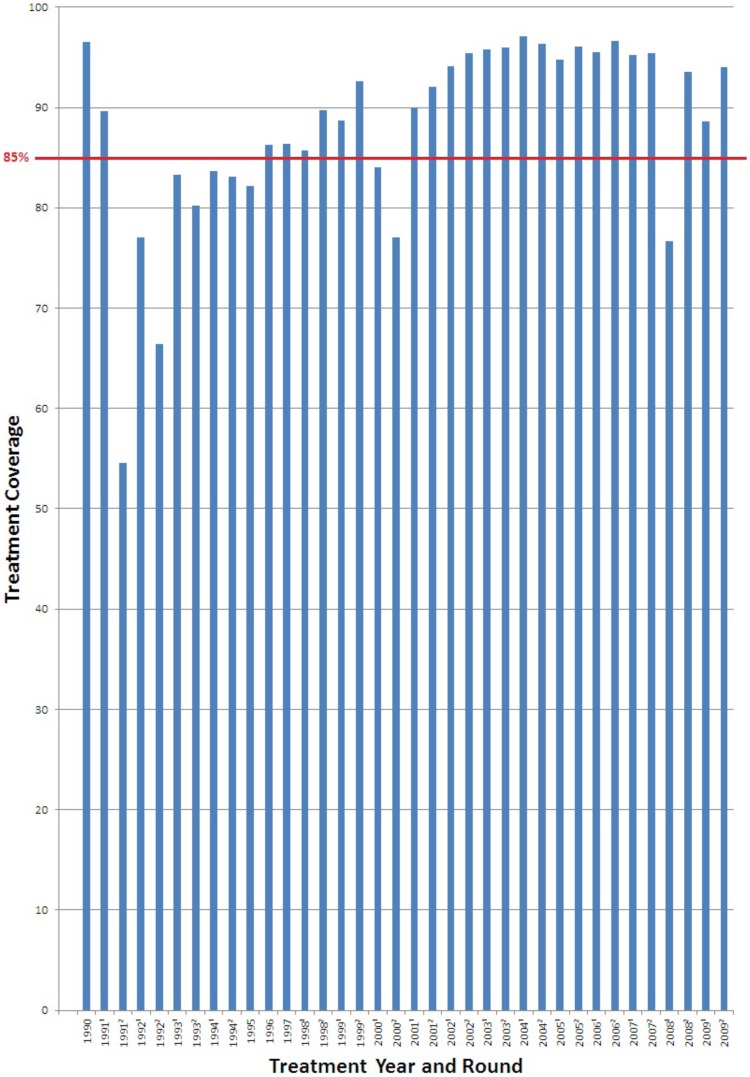
Drug distribution coverage for the Onchocerciasis Program. Percentage (%) of eligible people treated every 6 months from 1991–2009. Line indicates a 85% coverage level.

Coverage rates were estimated from the detailed medical records kept on each resident in the treatment areas and expressed as % of the eligible individuals in the village. Throughout the period of mass drug administration (1991–2009) ivermectin was distributed in 35 rounds to eligible residents of the major endemic area (Figure 2) twice a year, except for 1995–7 when only one treatment was provided. In the majority of the 18 years during which treatment was given, at least one of the round of treatment exceeded this 85% level, and in most cases this level was achieved both times during the year. In the 12 year period, from 1998 to 2009, 24 bi-annual treatment rounds were administered, with a coverage of at least 85% of the eligible population in all but 2 of them (i.e. the second round in 2000–%, and in first round 2008–74%).

### Sample collection

Black flies were collected at seven community sites in the Esmeraldas Province of Ecuador (Figure 1 & Table 1). Flies were collected using standard methods in the months of April-June, 2012; these months correspond to the peak of transmission. Parous females were not separated from nulliparous insects as these samples were used in a pool screening assay. Collections were carried out from 0800-1700 each day for 8 days per month. Flies were collected for 50 minutes each hour, allowing a 10-minute break to the collectors during each hour. The collected flies were sorted according to species (*S. exiguum* and *S. quadrivitatum*), divided into pools containing a maximum of 50 insects from a given species per pool, placed in isopropanol and stored at room temperature until analyzed by PCR.

**Table 1:** Entomological assessment 1995, 2000, 2004 and 2008, in the major collection sites located in three major river systems in the onchocerciasis endemic zone of Ecuador.

River	Community	1995		2000		2004		2008	
		No.	IR (CI)	No.	IR (CI)	No.	IR (CI)	No.	IR (CI)
Río Cayapas	Corriente Grande	ND	-	23500	2.1 (0.7–5.0)	12700	0 (0–0.30)	14477	0 (0–0.26)
	El Tigre	2550	72.7 (42.0–116.0)	17000	9.0 (4.5–6.1)	10100	6.0 (2.05–13.3)	10180	0 (0–0.38)
	San Miguel	3550	65.0 (39.0–101.0)	9600	1.1 (0.03–6.4)	12500	0.8 (0.02–4.1)	11863	0 (0–0.32)
Río Santiago	Playa de Oro	ND	-	10250	0 (0–0.37)	9500	0 (0–0.40)	ND*	-
	Guayabal	ND	-	9950	0 (0–0.38)	10000	0 (0–0.38)	ND*	-
	Angostura	ND	-	9700	0 (0–0.40)	11400	0 (0–0.34)	ND*	-
Río Canande	Naranjal	ND	-	20450	0.5(0.01–2.0)	26050	0 (0–0.15)	12398	0 (0–0.31)

No. =  Number of black flies analyzed; IR =  infectivity rate in fly heads (per 2000); CI =  Confidence Interval; ND =  Not done;

* =  treatment was stopped in this particular river system prior to this date.

For serology, samples were taken from children under the age of 17 from four sentinel communities (211 individuals in 2001; 519 in 2008) and eight extra-sentinel communities (318 individuals in 2006; 762 in 2007) for a total of 1941 individuals tested.

### Entomological (PCR) testing

Heads and bodies from the collected flies were separated by freezing, agitation and separation through a 25 mesh sieve, as previously described [15]. DNA was prepared from the separated heads and bodies by proteinase K digestion, organic extraction and adsorption to a silica matrix, as previously described [16]. Pools were processed in groups consisting of 50 flies in each pool; one sham extraction served as an internal negative control. The resulting DNA preparations were used as templates in a PCR assay targeting an *O. volvulus* specific repeated sequence (O-150 PCR), as previously described [15], [16]. PCR products were detected by PCR-ELISA. Pools producing ELISA values which were equal to or greater than the mean plus three standard deviations of the values obtained from 10 negative control wells run on each plate were considered to be putatively positive for *O. volvulus* DNA. Putatively positive DNA samples were re-tested in an independent PCR procedure and samples that were positive in both assays classified as confirmed positives. Pools of bodies were initially screened, as bodies contain early stage larvae (microfilarial and L_2_ stages) and are the most sensitive indicator of parasite - vector contact. The prevalence of flies containing immature stages is 2 fold higher than the prevalence of flies containing infective stage larvae (L_3_) in *S. exiguum* and 20 fold higher in *S. quadrivitatum* [17]. DNA from head pools were screened if evidence for parasite-vector contact was observed in the screens of the body pools in order to obtain an estimate of the prevalence of flies containing L_3_. The upper bound of the 95% confidence interval for the prevalence of flies carrying *O. volvulus* parasites was calculated using the Bayesian algorithm of Poolscreen v 2.0. In undertaking these calculations, the mean number of L_3_s per infective fly was taken as 1, as reported to be the case in areas subject to effective control measures [16].

### Serological studies

The Ov-16 ELISA assay uses a recombinant antigen of *O. volvulus* [18], [19], [20] to measure prevalence of IgG4 antibodies against the corresponding native antigen as a surrogate measure of exposure to the parasite. Blood spots for the Ov16 assay were collected in July 2007 and from December 2010 to January 2011. In both studies, sterile techniques were used to collect finger prick blood onto 5×5 cm area of filter paper (Whatman type 2). The saturated blood spots were dried, individually wrapped and transported at 4°C to the laboratory where they were stored at -20°C for further analysis. In the laboratory, sera were eluted from filter paper punches and used in standard ELISA assay as previously described [21]. A standard curve was used with each ELISA plate to identify positive samples and permit comparisons between plates and over time. The cut-off was chosen as 40 arbitrary units by identifying the value that optimized both sensitivity and specificity for Latin American and African positive and negative samples as previously reported [20], [21]. A sample is reported positive only after a second independent positive repeat test.

### Statistical methods

Poolscreen algorithm Versions 2.0 [17] was used to analyse the data and to calculate the infectivity rate (i.e. the prevalence of infection with L3), and associated 95% CI (Confidence Interval), in the vector populations. No statistics were required to compare serology results as all test were below the value set as positive [20], [21].

## Results

### PCR detection of infected flies

Samples taken in 1995 from two collection sites on the Rio Cayapas in the hyper-endemic zone showed infectivity rates (IR) of 72.7 and 65.0 (Table 1); these sites, and subsequently others, showed a reduction in IR for 2000 and 2004. In 2009 these and all tested sites were negative for *O. volvulus* DNA in the flies collected. The IR at each site declined in each additional year tested. In 2000 no evidence of the presence of infected flies was seen in any of the site tested in the Rio Santiago and testing was not continued on this river in subsequent years. Negative results were also achieved in the other two river systems in 2004 and 2008, although Rio Cayapas had two sites with low levels of IR in 2004, both of which became negative in 2008. All sites were negative by 2008.

### Antibody presence in young residents

Young residents under the age of 15 from 14 different communities in the major river system were tested during the period 2001–2008 on four different occasions; at no time was any individual found to be positive for anti-Ov-16 antibodies (Tables 2 and 3).

**Table 2:** The prevalence of anti-*Onchocerca volvulus* antibodies (Ov-16 antigen) in 1–15 year old children resident in the community collection sites in the onchocerciasis endemic area of Ecuador.

Date	River	Community	Rounds	Age (years)	Total number present	No. examined (%)	No. with Antibody positive (%)
2001	Río Santiago		15	15o	NA	211 (NA)	0
2008	Río Cayapas	Corriente Grande	32	2–15	127	116 (91.3)	0
		El Tigre	24	2–15	94	74 (78.7)	0
		San Miguel	24	2–15	90	82 (91.1)	0
		TOTAL	24–32	2–15	311	272 (87.5)	0
2008	Río Canande	Naranjal	29	2––15	298	247 (82.9)	0

Samples taken in 2001 and 2008.

Rounds  =  The number of rounds of treatment received; Total number present  =  Total number of children within the tested age range present in the community; NA =  not available

**Table 3:** The prevalence of anti-*Onchocerca volvulus* antibodies (Ov-16 antigen) in 1-16 year old children resident in various communities within the onchocerciasis endemic area of Ecuador.

DATE	RIVER	COMMUNITY	Rounds	Age (years)	Total number present	No. examined (%)	No. antibody positive (%)
2006	Río Cayapas	Agua Blanca	28	1–10	196	181 (92.3)	0
		Callemansa	28	1–10	151	137 (90.7)	0
	TOTAL				347	318 (91.6)	0
2007	Río Zapallo	Agua Salada	30	1–16	72	72 (100.0)	0
		Pichiyacu	30	1–16	92	89 (96.7)	0
		Herradura	30	1–16	111	110 (99.1)	0
		Playa Grande	30	1–16	39	39 (100.0)	0
	TOTAL				314	310 (98.7)	0
2007	Río Onzole	Colón de Onzole	23	1–16	322	314 (97.5)	0
		Capulí	23	1–16	133	133 (100.0)	0
		Las Pavas	23	1–16	5	5 (100.0)	0
	TOTAL				460	452 (98.3)	0

Samples taken in 2006 and 2007.

Rounds  =  The number of rounds of treatment received; Total number present  =  Total number of children within the tested age range present in the community; NA =  not available.

## Discussion

Nodulectomy, the approach originally used to control the increasingly prevalent clinical onchocerciasis seen in this endemic area before the introduction of ivermectin, was found to be singularly unsuccessful, with the prevalence of clinical disease, including severe eye problems, increasing significantly during the period from 1980 to 1989 [5], [12]. The introduction of the administration of ivermectin twice a year has had a remarkable effect on reducing all aspects of this disease complex and the findings described in this present paper indicate that the focus is moving successfully towards elimination of transmission of the infection.

The international community has developed a series of metrics, relying upon entomological and epidemiological indicators to determine when transmission had been suppressed [13]. Once transmission is determined to have been interrupted, mass drug distribution can cease. Three years after mass drug distribution ended, a post-treatment surveillance (PTS) should be undertaken, which uses entomological indicators to look for evidence of recrudescence of transmission [13]. If no evidence for ongoing transmission is uncovered during the PTS, it can be concluded that transmission had been eliminated. Thus these guidelines described the stages and testing required for an endemic area, be it a country or a site within a country, to be able to claim elimination. Essentially there are two major steps a program must make to achieve formal verification that transmission is ceased: first is the cessation of treatment based on evidence that vectors no longer carry the parasite, and the second is the maintenance of this interrupted state for three years (the post-treatment phase). The data presented in this paper fulfills the first phase of these requirements.

Despite the severity of the disease in the Ecuadorian onchocerciasis focus, and the voracity of the vector in this region, the strategy of high coverage and great emphasis on contact and communication with the affected communities has produced very satisfactory results. This success, achieved with arguably the most severe focus of onchocerciasis in Latin America, emphasizes the likelihood that the strategy of semiannual treatments will be effective in this region, and probably can also be successful in a number of foci in Africa and Yemen. Ecuador also faced a number of challenges that are to be expected with an isolated disease focus including the possibility of migration of infected people out of the focus and the attendant potential of establishment of new foci. This possibility was carefully monitored in the Ecuadorian programme and treatments carried out in any possible for areas outside the main focus, such as is shown in Figure 1, where three sites outside the main three river focus were monitored, and one tested in this present reported data. It is most important in establishing the elimination of onchocerciasis from the country as a whole to consider the question of dissemination of infection through population migration.

The first program to implement an ivermectin based elimination strategy for onchocerciasis was the Onchocerciasis Elimination Programme of the Americas (OEPA). OEPA's strategy was to provide semi-annual treatments with ivermectin at a coverage rate ≥85% of all eligible at risk individuals residing in the 13 foci of onchocerciasis in the six endemic countries in Latin America. By ensuring high coverage rates in the eligible population over a period of several years, it was believed that transmission of the parasite could be suppressed for a long enough period that the parasite population would eventually be pushed below the transmission breakpoint, and the parasite population would become locally extinct. The success of this approach is now being seen in most of the Latin American foci with Colombia being the first country to fulfill criteria for verification of elimination and others expected to follow in the next few years.

Arguably the most important lesson emerging from the success here is that high coverage with twice a year administration of ivermectin can successfully, albeit over a relatively long period of time, eliminate transmission of this infection. The fact that the main vector in this area, *S. exiguum*, is a very efficient insect in terms of promoting *O. volvulus* infection suggests that the success seen here in Ecuador may indeed be able to be translated from this relatively small focus to the more challenging locations in Africa.

The data presented here were in concordance with the guidelines established by the World Health Organization. In addition, clinical and pathological monitoring by the programme has supported the basic entomological and serological results. These findings led to the cessation of mass drug treatment with ivermectin in the Ecuadorian onchocerciasis focus, and the entry of the programme into post treatment surveillance phase. The maintenance of this status of interrupted transmission is needed for a further three years to complete successful elimination of this infection from the country [13].
